# Plasma Amino Acids and Residual Hypertriglyceridemia in Diabetic Patients Under Statins: Two Independent Cross-Sectional Hospital-Based Cohorts

**DOI:** 10.3389/fcvm.2021.605716

**Published:** 2021-05-31

**Authors:** Shuang Wang, Yun-Feng Cao, Xiao-Yu Sun, Mo Hong, Zhong-Ze Fang, Hui-Huan Luo, Huan Sun, Ping Yang

**Affiliations:** ^1^Cardiology Department, China-Japan Union Hospital of Jilin University, Changchun, China; ^2^Jilin Provincial Molecular Biology Research Center for Precision Medicine of Major Cardiovascular Disease, Changchun, China; ^3^Jilin Provincial Cardiovascular Research Institute, Changchun, China; ^4^Key Laboratory of Liaoning Tumor Clinical Metabolomics, Jinzhou, China; ^5^RSKT Biopharma Inc, Dalian, China; ^6^Department of Toxicology and Sanitary Chemistry, School of Public Health, Tianjin Medical University, Tianjin, China

**Keywords:** hypertriglyceridemia, amino acids, statins, type 2 diabetes, cardiovascular diseases

## Abstract

**Objective:** The objective of the study was to investigate the relationship of amino acid metabolism with hypertriglyceridemia in diabetic patients under statins free of prior cardiovascular diseases.

**Methods:** Two independent cross-sectional hospital based cohorts, i.e., Liaoning Medical University First Affiliated Hospital (LMUFAH, *n* = 146) and the Second Affiliated Hospital of Dalian Medical University (SAHDMU, *n* = 294) were included in the current analysis. Hypertriglyceridemia was defined as triglyceride ≥1.7 mmol/L, and well-controlled LDL-C was defined as <2.6 mmol/L. The adjusted ORs (95% CI) of circulating metabolic measures for hypertriglyceridemia were assessed using logistic regression. Pooled results of metabolites with the same direction of association in both cohorts were combined using inverse variance-weighted fixed-effect meta-analysis. Difference of identified metabolites in patients with and without hypertriglyceridemia were also obtained in the context of LDL-C.

**Results:** Patients, 86 and 106, were with hypertriglyceridemia in LMUFAH and SAHDMU, respectively. We observed that elevated alanine, asparagine, leucine, and valine were consistently associated with increased hypertriglyceridemia in both cohorts. In fixed-effect pooled analysis, the OR (95% CI) per SD increase was 1.71 (1.32–2.20) for alanine, 1.62 (1.20–2.19) for asparagine, 1.64 (1.22–2.20) for leucine, and 1.62 (1.22–2.13) for valine (all *P* values ranged from 0.0018 to <0.0001); adjusting for C-peptide attenuated effect sizes of Ala, Leu, and Val for hypertriglyceridemia. The difference were robust in groups with well- or bad-controlled LDL-C.

**Conclusion:** Among 23 amino acids, alanine, asparagine, leucine, and valine were positively associated with increased residual risk of hypertriglyceridemia in diabetic patients with statin treatment.

## Introduction

Hypertriglyceridemia (HTG) is defined as triglyceride ≥1.7 mmol/L. Recent evidence suggest that HTG, as a risk factor for cardiovascular diseases (CVD), is independent of low-density lipoprotein cholesterol (LDL-C), and high-density lipoprotein cholesterol (HDL-C) (1–3). Patients with type 2 diabetes (T2D) are more likely to have dyslipidemia and elevated CVD risk (4, 5). For the prevention of CVD, lipid-lowering agents are generally recommended for people with diabetes (6, 7). As first-line lipid-lowering treatment, statins can markedly lower LDL-C by blocking synthesis of cholesterol in the liver, and reduce CVD risk subsequently (8, 9). By contrast, statins' effect on triglyceride (TG) is moderate, with high dose of statins reducing 20–40% of TG (10, 11). Substantial residual HTG contributes to increased risk of CVD even among diabetics with well statin-controlled LDL-C (8, 12, 13). High dosage of statins can induce a range of side effects related to rhabdomyolysis, cognitive impairment, hepatotoxicity, and so on (14, 15). So instead of intensive use of statins, combined medication may be a better option for patients with poor response to current statin therapy. Additional efforts are needed to identify these subjects and explore potential new targets for their TG lowering.

Liquid chromatography-mass spectrometry (LC-MS) enables high-throughput analysis of metabolites and provides novel insight into metabolic pathway discovery (16). Amino acids are important components for protein synthesis and play significant roles in a number of physiological processes including energy production, inflammation, signaling, insulin resistance, redox, and so on (17–20). In this connection, amino acids were identified as new biomarkers of chronic conditions such as diabetes, CVD, and obesity (16, 21–23). Metabolites have the potential of clinical utility with regard to assessing therapeutic effectiveness and response (24). Given that both TG and amino acids are closely linked to obesity and CVD development in T2D, we would like to know whether plasma-free amino acids can identify diabetic subgroups that can or cannot respond well to the TG-lowering property of statins and perform as potential novel therapeutic targets.

In this study, we aimed to assess (1) if amino acids are associated with residual hypertriglyceridemia in diabetic patients with statin treatment and (2) if the relationships remain in the context of LDL-C. Two independent cross-sectional hospital-based cohorts were included in the current analysis.

## Materials and Methods

### Study Populations

The study involved individuals from two cross-sectional hospital-based cohorts in China. Details of the two cohorts are as follows:

#### Liaoning Medical University First Affiliated Hospital

**Figure 1:**
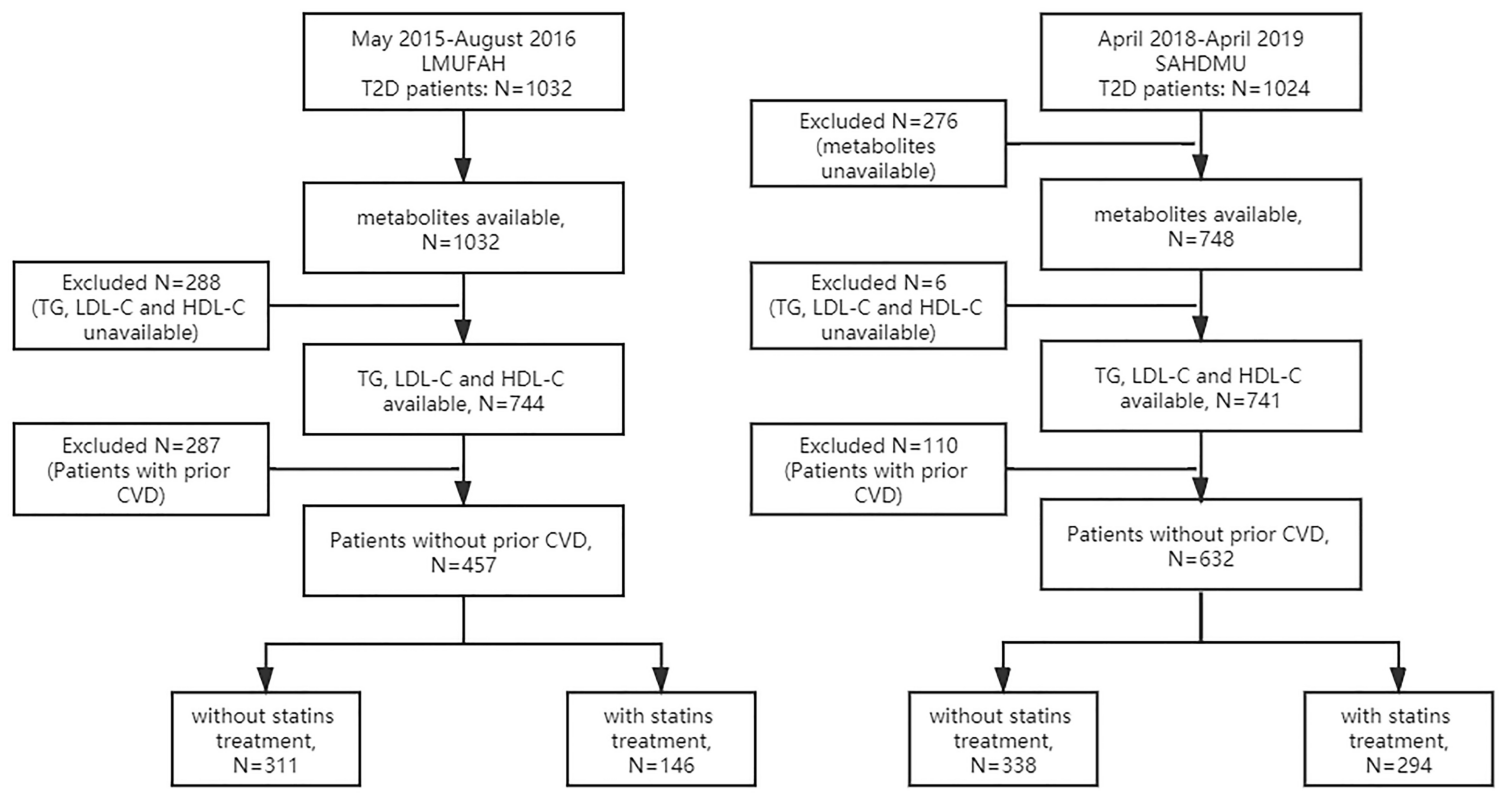
Flow diagram of selection of subjects included in this study. LMUFAH, Liaoning Medical University First Affiliated Hospital; SAHODMU, the Second affiliated hospital of Dalian Medical University; T2D, type 2 diabetes; TG, triglyceride; LDL-C, low-density lipoprotein cholesterol; HDL-C, high-density lipoprotein cholesterol; CVD, cardiovascular diseases, including coronary heart disease and stroke.

The details of this cohort are described elsewhere (25). Briefly, from May 27, 2015 to August 3, 2016, serum metabolites were quantified from 1,032 consecutive diabetic patients. Clinical information was retrospectively extracted from electronic medical records. Among 1,032 patients, 288 patients were excluded due to lack of complete information on TG, LDL-C, and HDL-C. Of the remaining 744 patients, 287 with prior coronary heart disease (CHD) and stroke were excluded. Among the remaining 457 patients, 146 were with statin treatment, and 311 were without statin treatment. Finally, the main analysis included 146 individuals (1) with complete information on TG, LDL-C, and HDL-C, (2) with complete metabolomic profile, (3) without prior CHD and stroke, and (4) with statin treatment (Figure 1). The protocol of the study was approved by the Ethics Committee for Clinical Research of Liaoning Medical University First Affiliated Hospital (LMUFAH). Informed consent was waived due to the nature of the retrospective study, which was in accordance with the Helsinki Declaration of 1964 and its later amendments.

#### The Second Affiliated Hospital of Dalian Medical University

From April 2018 to April 2019, a total of 1,024 consecutive diabetic subjects were admitted into The Second Affiliated Hospital of Dalian Medical University (SAHDMU) and agreed to participate in this research. As in LMUFAH, electronic medical records were collected. Serum metabolites were quantified in 748 of them. Subsequently, six patients were excluded due to lack of complete information on TG, LDL-C, and HDL-C. Of the remaining 741 patients, 110 with prior CHD and stroke were excluded. The remaining 631 patients included 294 with statin treatment and 338 without statin treatment. The final analysis was limited to 294 individuals (1) with complete information on TG, LDL-C, and HDL-C, (2) with complete metabolomic profile, (3) without prior CHD and stroke, and (4) with statin treatment (Figure 1). The Ethics Committee for Clinical Research of SAHDMU approved the ethics of the study, and all the participants provided informed written consent.

### Data Collection and Definitions

T2D was diagnosed by the 1999 World Health Organization's criteria (26) or treated with antidiabetic drugs. CHD was defined as having a history of angina with abnormal electrocardiogram or on stress test, myocardial infarction, angina coronary artery bypass graft surgery, or angioplasty; stroke was defined as non-fatal subarachnoid hemorrhage, intracerebral hemorrhage, or other unspecified intracranial hemorrhage and ischemic stroke; HTG was defined as triglyceride ≥1.7 mmol/L; treatment goals was <2.6 mmol/L for LDL-C, <1 mmol/L in male or <1.3 mmol/L in female for HDL-C, and <7% for glycated hemoglobin (HbA1c) (6).

Other available information in the current analysis included age, sex, current smoking, current drinking, body mass index (BMI), systolic blood pressure (SBP), fasting C-peptide (only available in SAHDMU), duration of diabetes, diabetic nephropathy (DN), diabetic retinopathy (DR), and use of antidiabetic agents. BMI was calculated as the ratio of weight in kilograms to height squared in meters; DR was evaluated by bilateral retinal photography and was defined as the presence of microaneurysms, retinal hemorrhages, soft exudates, hard exudates, or vitreous hemorrhage; DN was defined as persistent albuminuria, progressive reduction in glomerular filtration rate, and hypertension judged by clinicians (27); antidiabetic agents included insulin and other oral antidiabetic agents.

### Amino Acid Quantification

Details of the metabolomics assessment method were published previously (28). Briefly, 8 h of fasting blood sample was collected at admission. A total of 23 amino acids, i.e., alanine (Ala), asparagine (Asn), leucine (Leu), phenylalanine (Phe), tryptophan (Trp), tyrosine (Tyr), valine (Val), arginine (Arg), glycine (Gly), proline (Pro), threonine (Thr), citrulline (Cit), glutamine (Gln), histidine (His), lysine (Lys), methionine (Met), serine (Ser), ornithine (Orn), glutamate (Glu), aspartate (Asp), piperamide (Pip), cysteine (Cys), and homocysteine (Hcy), were detected via LC-MS. AB Sciex 4000 QTrap system (AB Sciex, Framingham, MA, USA) was used to conduct direct injection MS metabolomic analysis. Analyst v1.6.0 software (AB Sciex) was used for data collection. ChemoView 2.0.2 (AB Sciex) was used for data preprocessing. Isotope-labeled internal standard samples were purchased from Cambridge Isotope Laboratories (Tewksbury, MA, USA). Standard samples of the amino acids were purchased from Chrom Systems (Grafelfing, Germany).

### Statistical Analysis

Characteristics of participants in two cohorts were described and compared according to TG levels (TG <1.7 vs. ≥1.7 mmol/L). Continuous data with normal distribution were expressed as the mean ± standard deviation (SD), while data with skewed distribution were presented as median with interquartile range (IQR). Normality was tested by checking the Q-Q plot. Categorical data were presented as *n* (%). Differences between subjects with optimal TG and HTG were compared by Student's *t*-test (or Mann–Whitney *U* test when appropriate) for continuous variables and Chi-square test (or fisher test if appropriate) for categorical variables. False discovery rate (FDR) was calculated for multiple comparisons of 23 amino acids and *q* < 0.05 was defined as statistically significant.

According to FDR, four amino acids with the same direction of association in both cohorts were selected into further analysis. The ORs (95% CI) of four circulating metabolic measures with HTG were assessed using logistic regression. Before introducing into regression models, all metabolites were scaled to SD concentrations separately for each cohort. A structured adjustment scheme was used to control for confounders: model 1, adjusted for age and sex; model 2, adjusted for variables in model 1 plus BMI, duration of diabetes, and DN; and model 3, adjusted for variables in model 2 plus HDL-C, LDL-C, and HbA1c. To explore whether insulin action mediated part of the effects of amino acid metabolism, we also adjusted for variables in model 3 plus C-peptide in model 4. Pooled results from individual cohorts were combined using inverse variance-weighted fixed-effect meta-analysis.

Pearson or Spearman correlation was used to calculate the coefficients within selected metabolites and clinical biochemical parameters, i.e., four amino acids, TG, HDL-C, LDL-C, HbA1c, and C-peptide.

To explore associations between amino acids and residual HTG in the context of LDL-C, we also repeated Student's *t*-test (or Mann–Whitney *U* test when appropriate) in subjects with LDL-C <2.6 mmol/L and LDL-C ≥2.6 mmol/L separately. Box plot was used to exhibit the distribution and difference visually. The same procedures were conducted in groups with either normal or abnormal HbA1c, and groups with either normal or abnormal HDL-C.

Given that we did not collect a specific dose of statins, to eliminate possible impact of statins on association between amino acids and TG, we also compared metabolic profile in subjects without statin treatment according to TG levels and obtained ORs (95% CI) using logistic regression as well.

All analyses were performed using SAS version 9.4 (SAS institute Inc., Cary, NC, USA) and R version 3.6.2.

## Results

### Characteristics of the Study Population

The characteristics of the study participants are shown in Table 1. Characteristic distribution was different in two cohorts. In LMUFAH, among 146 subjects, 86 of them were with HTG. The 146 patients had a mean age of 55.4 (SD: 11.5) years and median duration of T2D of 5 (IQR: 0–10) years. Compared with patients with normal TG, patients with HTG had higher BMI and LDL-C. In SAHODMU, 106 of 294 subjects were with HTG. Mean age of 294 patients was 59.0 (SD: 12.2) years, and median duration of diabetes was 9 (IQR: 3–16) years. Compared with patients with normal TG, patients with HTG was younger and had lower HDL-C, LDL-C, and higher fasting C-peptide. Difference of other characteristics were not statistically significant in each cohort (Table 1).

**Table 1:** Clinical and biochemical characteristics of participants in two cohorts according to TG levels.

	**LMUFAH**			**SAHODMU**		
	**TG <1.7 mmol/L**	**TG ≥ 1.7 mmol/L**	***P***	**TG <1.7 mmol/L**	**TG ≥ 1.7 mmol/L**	***P***
*N*	60	86		106	188	
TG, mmol/L	1.13 (0.83–1.35)	2.48 (2.03–3.20)	<0.0001	1.11 (0.88–1.42)	2.68 (2.04–3.87)	<0.0001
Age, years	56.0 ± 11.2	55.0 ± 12.2	0.6047	62.2 ± 11.0	57.8 ± 12.7	0.0011
Sex, male	33 (55.0)	48 (55.8)	0.9224	62 (58.5)	89 (47.3)	0.0663
Duration of diabetes, years	5 (0–11)	5 (0–10)	0.8364	11 (4–17)	8 (2–15)	0.0754
Body mass index, kg/m2	24.4 ± 3.4	26.3 ± 3.4	0.0016	26.6 ± 4.5	27.0 ± 3.5	0.3727
Current smoking	25 (41.7)	33 (38.4)	0.6890	25 (23.6)	39 (20.7)	0.5710
Current drinking	20 (33.3)	33 (38.4)	0.5333	10 (9.4)	22 (11.7)	0.5488
Systolic blood pressure, mmHg	140.0 ± 24.2	138.0 ± 22.5	0.6073	152.2 ± 19.8	148.9 ± 21.3	0.1947
HbA1c, %	10.3 ± 2.3	9.8 ± 2.2	0.1774	8.9 ± 2.2	9.2 ± 2.1	0.1747
≥7.0	47 (94.0)	70 (90.9)	0.7389	83 (78.3)	162 (86.2)	0.0822
HDL-C, mmol/L	1.17 ± 0.36	1.09 ± 0.29	0.1886	1.41 ± 0.42	1.08 ± 0.31	<0.0001
<1.00 in male or <1.30 in female	35 (58.3)	55 (64.0)	0.4920	23 (21.7)	115 (61.2)	<0.0001
LDL-C, mmol/L	3.13 ± 0.96	3.56 ± 1.04	0.0118	2.97 ± 0.80	2.68 ± 0.97	0.0062
≥2.60	42 (70.0)	72 (83.7)	0.0486	81 (76.4)	103 (54.8)	0.0002
Fasting C-peptide, ng/ml	-	-	-	1.12 (0.81–1.55)	1.75 (1.26–2.34)	0.0002
Antidiabetic agents	60 (100.0)	83 (96.5)	0.2688	101 (95.3)	176 (93.6)	0.6147
Diabetic nephropathy	23 (38.3)	21 (24.4)	0.0714	51 (48.1)	87 (46.3)	0.7619
Diabetic retinopathy	14 (23.3)	17 (19.8)	0.6042	26 (25.2)	58 (31.2)	0.2868

*TG, triglyceride; HDL-C, high-density lipoprotein cholesterol; LDL-C, low-density lipoprotein cholesterol; HbA1c, glycated hemoglobin; LMUFAH, Liaoning Medical University First Affiliated Hospital; SAHODMU, the Second affiliated hospital of Dalian Medical University.*

*Data are mean ± standard deviation, median (IQR), or n (%).*

*P values were derived from independent-samples Student t test for normally distributed variables, Mann-Whitney U test for skewed distributions, Chi-square test (or fisher test if appropriate) for categorical variables. P < 0.05 was defined as statistically significant*.

### Differences in Individual Amino Acids According to Triglyceride Levels

We observed four metabolites, i.e., Ala, Asn, Leu, and Val, demonstrating significant associations (all FDRs < 0.05) in the same direction with HTG in both cohorts. Other amino acids were similar between patients with and without HTG (Table 2).

**Table 2:** Plasma amino acids levels in two cohorts according to TG levels.

	**LMUFAH**			**SAHODMU**		
	**TG <1.7 mmol/L**	**TG ≥ 1.7 mmol/L**	***q***	**TG <1.7 mmol/L**	**TG ≥ 1.7 mmol/L**	***q***
Ala, μmol/L	119.70 ± 37.28	136.70 ± 40.31	0.0109	164.90 ± 50.98	185.70 ± 57.04	0.0092
Arg, μmol/L	8.67 (5.24–15.01)	10.56 (5.77–16.65)	0.4471	3.36 (1.88–4.61)	2.80 (1.86–4.56)	0.8663
Asn, μmol/L	72.23 ± 19.55	84.12 ± 24.09	0.0219	69.60 ± 16.67	78.22 ± 25.54	0.0029
Asp, μmol/L	27.59 ± 12.17	29.86 ± 10.99	0.3966	25.79 ± 10.53	28.14 ± 12.30	0.3157
Cit, μmol/L	21.19 ± 6.94	20.91 ± 7.32	0.8926	24.67 (20.48–30.63)	22.17 (17.18–29.75)	0.0157
Cys, μmol/L	1.24 ± 0.58	1.31 ± 0.58	0.5591	1.33 (0.80–1.87)	1.29 (0.85–1.94)	0.8663
Gln, μmol/L	6.31 (4.71–8.71)	7.40 (5.79–9.14)	0.1734	8.50 (5.72–11.06)	7.97 (5.83–11.02)	0.8663
Glu, μmol/L	87.99 (75.81–102.94)	94.78 (83.50–111.10)	0.0843	129.08 (104.02–157.22)	129.58 (107.28–153.97)	0.8663
Gly, μmol/L	184.30 ± 72.09	210.10 ± 80.46	0.1592	165.10 (141.94–185.09)	162.88 (144.52–186.65)	0.3157
Hcy, μmol/L	7.91 (6.45–8.63)	7.72 (6.40–8.16)	0.3291	8.57 (8.02–9.27)	8.48 (8.01–9.17)	0.8663
His, μmol/L	41.28 (32.77–66.60)	47.31 (34.91–77.75)	0.2578	64.27 (42.37–93.25)	67.07 (42.16–99.20)	0.7181
Leu, μmol/L	120.00 ± 39.89	145 ± 48.52	0.0219	111.34 (95.16–126.09)	122.35 (106.21–148.89)	0.0023
Lys, μmol/L	130.30 ± 60.30	131.30 ± 59.29	0.9194	134.32 (99.20–173.80)	137.53 (86.14–191.94)	0.8663
Met, μmol/L	16.47 (13.99–21.43)	16.37 (14.21–21.10)	0.6456	14.80 ± 3.95	14.40 ± 5.14	0.8663
Orn, μmol/L	16.57 (12.44–23.93)	17.49 (12.95–22.57)	0.9194	11.24 (9.11–14.28)	11.53 (8.41–14.98)	0.8751
Phe, μmol/L	42.62 ± 12.05	47.34 ± 11.45	0.0823	40.38 ± 12.05	38.95 ± 11.19	0.7181
Pip, μmol/L	128.37 (98.83–182.87)	124.67 (92.52–153.26)	0.3966	191.60 ± 76.82	194.00 ± 94.09	0.8663
Pro, μmol/L	486.90 ± 178.80	520.70 ± 186.90	0.3966	423.40 ± 153.00	496.90 ± 166.20	0.0023
Ser, μmol/L	51.21 (42.53–63.04)	51.25 (44.69–67.80)	0.4112	45.53 ± 10.59	45.11 ± 12.07	0.8663
Thr, μmol/L	23.26 ± 7.18	25.46 ± 6.82	0.1592	24.54 ± 7.59	23.97 ± 7.46	0.8663
Trp, μmol/L	44.17 ± 12.47	48.39 ± 13.61	0.1592	39.38 ± 11.21	41.78 ± 12.91	0.3157
Tyr, μmol/L	43.86 (34.27–54.65)	46.71 (38.20–56.51)	0.2593	51.84 ± 15.04	52.81 ± 17.29	0.8663
Val, μmol/L	129.70 ± 33.28	147.20 ± 36.53	0.0284	141.60 ± 32.37	157.50 ± 43.92	0.0029

*TG, triglyceride; LMUFAH, Liaoning Medical University First Affiliated Hospital; SAHODMU, the Second affiliated hospital of Dalian Medical University; Ala, aanine; Asn, asparagine; Leu, leucine; Phe, phenylalanine; Trp, tryptophan; Tyr, tyrosine; Val, valine; Arg, arginine; Gly, glycine; Pro, proline; Thr, threonine; Cit, citrulline;Gln, glutamine; His, histidine; Lys, lysine; Met, methionine; Ser, serine; Orn, ornithine; Glu, glutamate; Asp, aspartate; Pip, piperamide, Cys, cysteine; Hcy, homocysteine.*

*Data are mean ± standard deviation, median (IQR), or n (%).*

*False discovery rate was calculated for multiple comparisons and q <0.05 was defined as statistically significant*.

**Figure 2:**
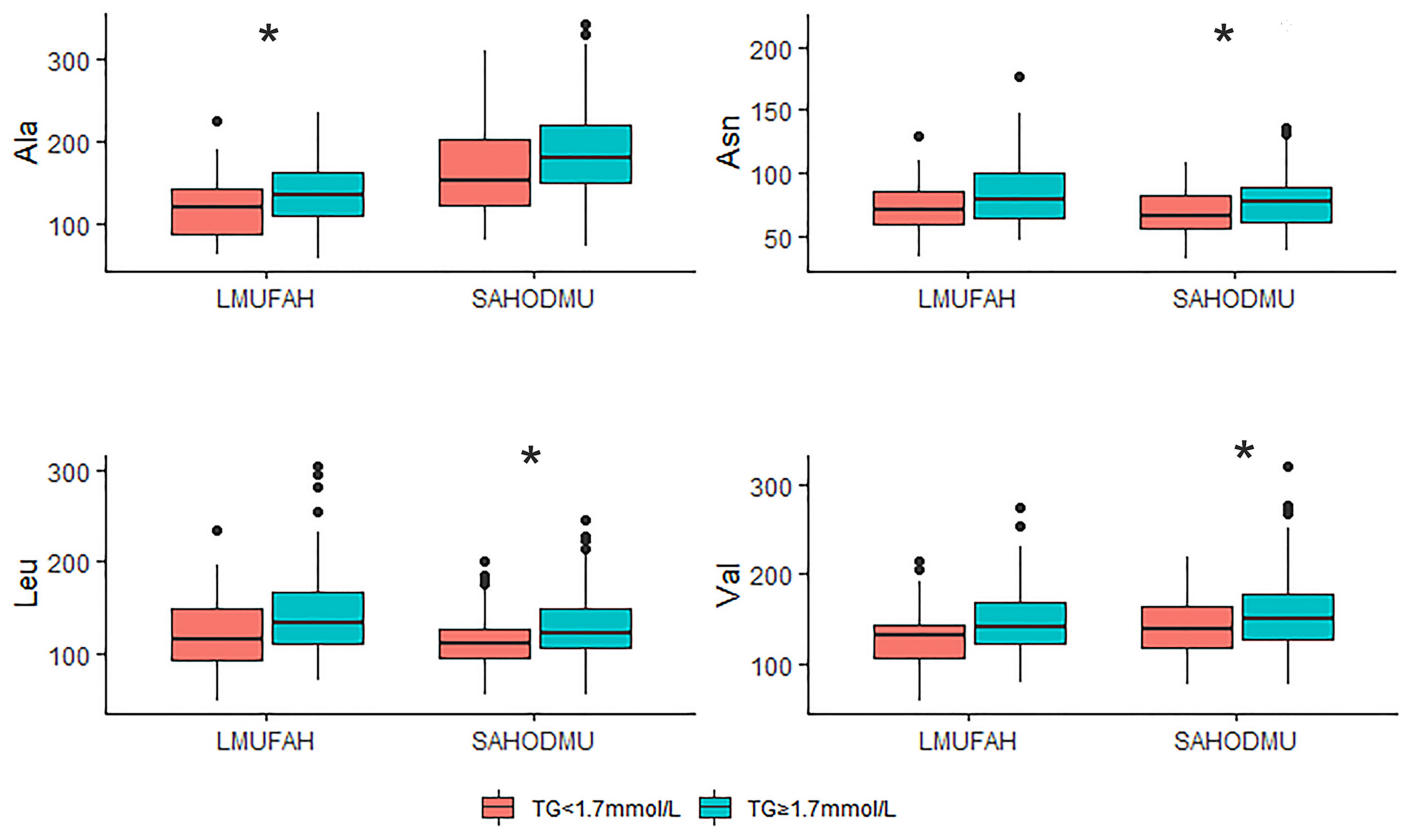
Plasma amino acids levels in patients with LDL-C <2.6 mmol/L according to TG levels. LDL-C, low-density lipoprotein cholesterol; TG, triglyceride; LMUFAH, Liaoning Medical University First Affiliated Hospital; SAHODMU, the Second affiliated hospital of Dalian Medical University; Ala, Alanine; Asn, Asparagine; Leu, Leucine; Val, Valine. *P < 0.05; **P < 0.01; ***P < 0.001.

**Figure 3:**
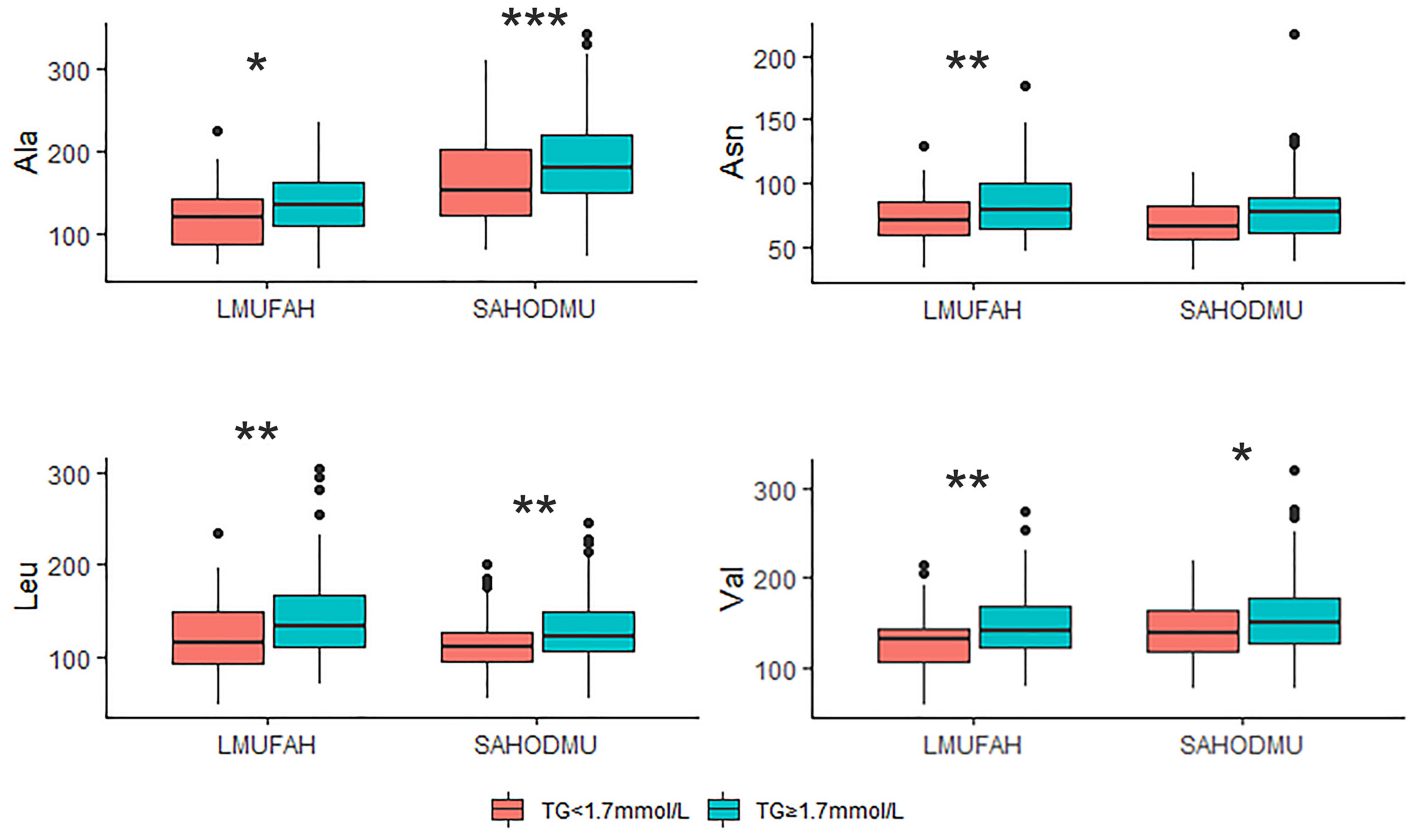
Plasma amino acids levels in patients with LDL-C ≥ 2.6 mmol/L according to TG levels. LDL-C, low-density lipoprotein cholesterol; TG, triglyceride; LMUFAH, Liaoning Medical University First Affiliated Hospital; SAHODMU, the Second affiliated hospital of Dalian Medical University; Ala, Alanine; Asn, Asparagine; Leu, Leucine; Val, Valine. *P < 0.05; **P < 0.01; ***P < 0.001.

For the subgroup with LDL-C <2.6 mmol/L, the directions of associations between amino acids and HTG were consist with the directions in the total group. However, in LMUFAH, the only difference in Ala was significant (*P* < 0.05). We speculated a non-significant difference in other three amino acids derived from a small sample size (*N* = 32); in SAHODMU, the difference in Asn, Leu, and Val were significant (*P* < 0.05), while the difference in Ala was not significant (Figure 2). For the subgroup with LDL-C ≥2.6 mmol/L, the directions of associations between amino acids and HTG were also consist with the directions in the total group, and all differences were significant except for Asn in SAHODMU (Figure 3).

Besides, in subgroups with different HbA1c or HDL-C, the differences in these four amino acids were robust too, although some of them were not statistically significant (majority with marginal significance) due to their small sample sizes (Supplementary Figures 1, 2).

### Correlations Within Selected Amino Acids and Clinical Biochemical Parameters

**Figure 4:**
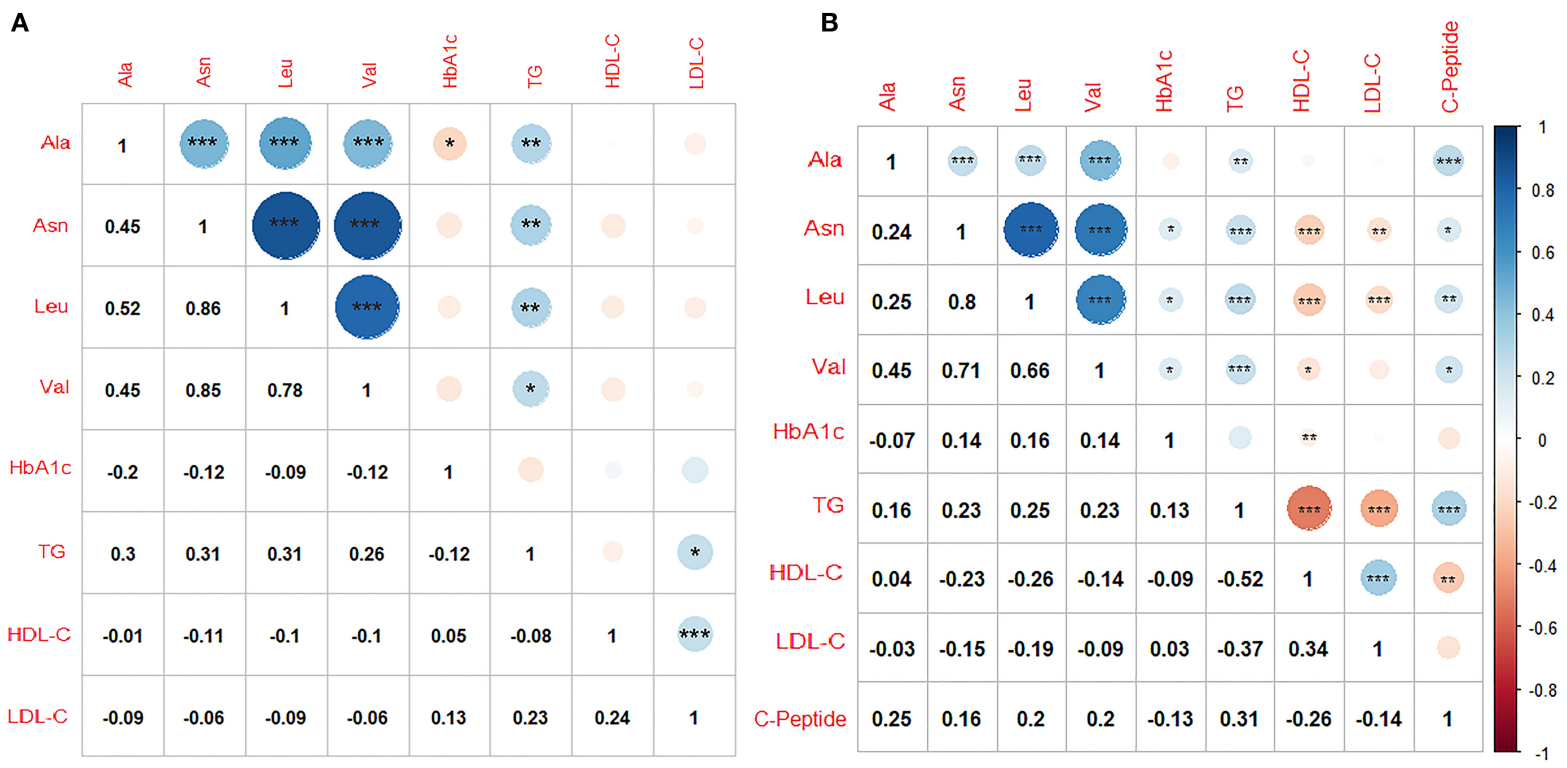
Correlations within metabolites and clinical biochemical parameters. **(A)** Liaoning Medical University First Affiliated Hospital; **(B)** the Second affiliated hospital of Dalian Medical University. Ala, Alanine; Asn, Asparagine; Leu, Leucine; Val, Valine; TG, triglyceride; LDL-C, low-density lipoprotein cholesterol; HDL-C, high-density lipoprotein cholesterol; HbA1c, glycated hemoglobin; Color blue, positive correlation coefficients; color red, negative correlation coefficients; Pearson or Spearman correlation was used to calculate the correlation coefficients; *P < 0.05; **P < 0.01; ***P < 0.001.

Amino acids were positively associated with each other and TG in both cohorts. There were only slight or no correlations between amino acids and HbA1c, with negative direction in LMUFAH and positive direction in SAHODMU. C-peptide was positively associated with amino acids (correlation coefficients ranged from 0.16 to 0.25) (Figure 4).

### Associations Between Selected Amino Acids and Hypertriglyceridemia

As shown in Table 3, the associations of these four metabolites with HTG risk remained after further adjustments of traditional risk factors, i.e., age, sex, BMI, duration of diabetes, and DN. In fixed-effect pooled analysis, the ORs (95% CI) of per SD increase were 1.49 (1.2–1.85) for Ala, 1.75 (1.35–2.26) for Asn, 1.74 (1.35–2.23) for Leu, and 1.58 (1.24–2.00) for Val (all *P* values ranged from 0.0003 to < 0.0001) (model 2). When further adjusted for HDL-C, LDL-C, and HbA1c, the pooled effect sizes were 1.71 (1.32–2.20) for Ala, 1.62 (1.20–2.19) for Asn, 1.64 (1.22–2.20) for Leu, and 1.62 (1.22–2.13) for Val (all *P* value ranged from 0.0018 to <0.0001). Adjusting for C-peptide attenuated associations in model 3, except for Asn (Ala, 7.9%; Leu, 2.7%; Val, 8.3%).

**Table 3:** Associations between amino acids and hypertriglyceridemia in two cohorts.

	**LMUFAH**		**SAHODMU**		**Pooled**	
	**OR (95% CI)**	***P***	**OR (95% CI)**	***P***	**OR (95% CI)**	***P***
**Model 1**						
Ala	1.59 (1.11–2.28)	0.0122	1.51 (1.16–1.97)	0.0024	1.54 (1.24–1.90)	<0.0001
Asn	1.98 (1.27–3.08)	0.0024	1.64 (1.19–2.25)	0.0025	1.75 (1.35–2.26)	<0.0001
Leu	1.99 (1.29–3.06)	0.0018	1.79 (1.31–2.46)	0.0003	2.37 (1.62–3.47)	<0.0001
Val	1.76 (1.19–2.59)	0.0047	1.51 (1.14–2.01)	0.0045	1.59 (1.27–2.01)	<0.0001
**Model 2**						
Ala	1.47 (1.02–2.13)	0.0399	1.50 (1.15–1.97)	0.0028	1.49 (1.20–1.85)	0.0003
Asn	1.77 (1.15–2.71)	0.0091	1.74 (1.25–2.42)	0.0011	1.75 (1.35–2.29)	<0.0001
Leu	1.73 (1.11–2.69)	0.0147	1.74 (1.29–2.35)	0.0003	1.74 (1.35–2.23)	<0.0001
Val	1.55 (1.03–2.32)	0.0344	1.59 (1.19–2.14)	0.0020	1.58 (1.24–2.00)	0.0002
**Model 3**						
Ala	1.60 (1.04–2.46)	0.0340	1.77 (1.29–2.41)	0.0004	1.71 (1.32–2.20)	<0.0001
Asn	1.91 (1.16–3.15)	0.0114	1.47 (1.01–2.15)	0.0456	1.62 (1.20−2.19)	0.0018
Leu	2.03 (1.18–3.49)	0.0105	1.50 (1.06–2.13)	0.0233	1.64 (1.22–2.20)	0.0010
Val	1.71 (1.05–2.77)	0.0309	1.57 (1.11–2.21)	0.0104	1.62 (1.22–2.13)	0.0008
**Model 4**						
Ala	-	-	1.63 (1.17–2.27)	0.0040	-	-
Asn	-	-	1.47 (0.99–2.21)	0.0595	-	-
Leu	-	-	1.46 (1.01–2.09)	0.0420	-	-
Val	-	-	1.44 (1.01–2.04)	0.0425	-	-

*TG, triglyceride; LMUFAH, Liaoning Medical University First Affiliated Hospital; SAHODMU, the Second affiliated hospital of Dalian Medical University; OR, odds ratio; CI, confidence interval; Ala, Alanine; Asn, Asparagine; Leu, Leucine; Val, Valine.*

*Logistic regression was used to obtain per standard deviation increased OR.*

*Model 1, adjusted for age and sex.*

*Model 2, adjusted for variables in model 1 plus body mass index, duration of diabetes and diabetic nephropathy.*

*Model 3, adjusted for variables in model 2 plus high-density lipoprotein cholesterol, low-density lipoprotein cholesterol and glycated hemoglobin.*

*Model 4, adjusted for variables in model 3 plus C-peptide*.

In diabetic patients without statin treatment, the associations between amino acids and HTG were in accordance with findings in the group with statin therapy, although the effect sizes were attenuated (Supplementary Tables 1, 2).

## Discussion

In this cross-sectional investigation in two independent hospital-based studies of targeted metabolomics and HTG risk, we identified four amino acid metabolites, including Ala, Asn, Leu, and Val, consistently associated with increased risk of HTG despite statin use. Besides, the associations were robust in the context of LDL-C, suggesting that abnormal amino acid metabolism contributed to residual HTG ignorant of LDL-C levels.

Although the relationships between amino acids and lipid abnormality were yet completely clarified, their associations have been investigated in many studies. Compared with healthy controls, patients with abnormal BMI or non-alcoholic fatty liver disease have profound perturbation of amino acid metabolism (29, 30). Several cross-sectional and longitudinal studies also showed that amino acid signature significantly predicted future hypertriglyceridemia in children (31, 32). Among these altered amino acids, branched-chain amino acids (BCAAs) was the most robust regarding linkage to lipid abnormality.

Generally, after absorption from the intestines, BCAAs are first transaminated to branched-chain keto acids (BCKAs) by branched-chain amino acid transaminase. Then BCKAs are oxidized by branched-chain α-keto acid dehydrogenase (BCKDH), the rate-limiting enzyme complex. Subsequently, downstream products with further enzymes were involved in metabolism and provide many physiological benefits via mechanisms such as regulating β-cell function and adipose tissue metabolism (33, 34). However, emerging evidence has revealed that impaired adipose BCAA catabolic pathway with suppressed BCKDH activity (i.e., accumulation of circulating BCAAs and BCKAs) can lead to β-cell dysfunction through mechanisms including chronic hyperactivation of mammalian target of rapamycin (mTOR) signaling, oxidative stress, and so on (18, 35). In this connection, insulin promotes storage of TG in adipose tissues and reduces circulating level and ectopic storage. Conversely, insulin resistance and deficiency in T2D accelerates lipolysis in adipocytes and excessive secretion of TG-rich lipoprotein such as very low-density lipoprotein cholesterol (VLDL-C) and LDL-C from the liver (5). Previous studies found that BCAA was positively associated with plasma TG in non-diabetic cohorts (31, 32, 36), which is consistent with our findings in the present diabetic group. Moreover, we also found that C-peptide, a byproduct of proinsulin and a good predictor of insulin resistance (37), only mediated partial effects of BCAA on TG. This finding is also in accordance with earlier prospective research in non-diabetic young to elderly population, where associations between BCAA and HTG remained significant even after controlling for insulin resistance (32, 38). Our study further emphasizes the complex pathological mechanisms of BCAA beyond insulin resistance.

Apart from BCAA, we also identify a positive association between Ala and TG as several previous studies in non-diabetes (30, 39). Ala plays a key role in the glucose–alanine cycle between tissues and the liver. Briefly, in muscle and other tissues, pyruvate accepts an amino group from glutamate through the action of alanine aminotransferase (ALT), forming alanine, and α-ketoglutarate. In fasting, Ala can also derive from muscle protein breaking down. The alanine enters the bloodstream and then the liver, where the ALT reaction takes place in reverse, and generates glucose subsequently (40). Thus, elevated Ala in the current study may be a marker of enhanced muscle glycolysis, muscle protein breaking down, or liver injury. In accordance with this assumption, impaired carbon metabolism, and liver function always accompany rising TG (41, 42).

Findings regarding Asn in our study was opposite to previous research: Takashina et al. classified 83 subjects with normal glucose tolerance as obese or non-obese, and as visceral obesity or non-visceral obesity, and analyzed correlations between 23 plasma amino acids and obesity. They found that obesity or visceral obesity was negatively associated with Asn (43). In a case-control study of Iranian adults, compared with 100 controls, 200 obese patients had lower levels of Asn (29). As we speculated before, the discrepancy may derive from population heterogeneity. Although Asn may perform as a protective factor in these two scenarios, accelerated Asn consumption leads to a decreasing Asn level, while activating asparagine synthetase gene (ASNS) may lead to increasing circulating Asn (25, 44). More investigations are warranted to clarify the difference.

Genetic and epidemiologic evidence have provided robust evidence for the causal role of HTG for CVD risk (2). In terms of lowering TG, fibrates, niacin, and fish oil have better performance than statins (10, 45). A combination of statins and other lipid-lowering agents was considered in mixed dyslipidemia, which raises some safety concerns. For example, niacin may increase the risk of diabetes (46); fibrates may compound rhabdomyolysis induced by statins (47); fish oil was proved to attenuate cardiovascular diseases and NAFLD in the general person; however, its effect in the diabetic group is controversial (48). More targets, especially in patients with diabetes, are required for TG management. In the present study, the adverse effects of amino acids on TG were not eliminated by statins, so amino acids may provide additional benefits beyond statins.

Our study has significant implications for clinical practice. As stated above, controlling cardiometabolic risk factors plays a central role in CVD prevention of patients with diabetes. TG management was recently recommended, whereas agents including statins reduced only partial HTG risk. Amino acids can be novel targets in diabetic subjects with statin therapy. Our study found associations between amino acids and residual HTG risk, which may have partially been mediate by insulin resistance, suggesting that patients with residual HTG may benefit from the regulation of amino acid metabolism. Agents targeting amino acids can be an option for combinations with statins. Besides, more intensive treatment on insulin resistance may also be recommended in the absence of hypoglycemia. Apart from clinical practice, our study also generated new hypotheses for basic science: First, the mechanism linking amino acids with TG requires more investigations. Second, lipid abnormalities may explain some links between amino acids and increased CVD risk in T2D.

There are several limitations in our study too. First, provided the nature of cross-sectional study design, the causal relationship cannot be established, and prospective cohorts are warranted. Second, we did not collect the dose and frequency of statins. Instead, we repeated the analysis in the subgroups without statin treatment. The associations between amino acid and HTG still existed. So the difference in amino acids metabolism between patients with and without HTG may not be derived from disparity of dose and frequency of statin therapy. Third, a large amount of observations have revealed that amino acid metabolism is often deregulated in diabetic patients. Diabetes may bias our finding. Nevertheless, a previous study in non-diabetic young to elder subjects also found robust relationships between amino acids and TG.

In conclusion, we detected positive associations between four amino acids, i.e., Ala, Asn, Leu, and Val, and residual HTG risk in patients with diabetes and statin treatment. Prospective researches are needed to confirm the findings, and experimental studies are needed to elucidate the underlying mechanism that will shed light on the prevention of HTG, subsequently, CVD in diabetes with statins.

## Data Availability Statement

The raw data supporting the conclusions of this article will be made available by the authors, without undue reservation.

## Ethics Statement

The protocol of the study was approved by the Ethics Committee for Clinical Research of LMUFAH and the Ethics Committee for Clinical Research of SAHDMU. Informed consent of LMUFAH was waived due to the nature of the retrospective study, which was in accordance with the Helsinki Declaration of 1964 and its later amendments. And all the participants in SAHDMU provided informed written consent.

## Author Contributions

PY, HS, and H-HL designed the study and put forward the idea. H-HL and SW analyzed the data, wrote the first draft, and revised the paper. Y-FC, X-YS, MH, and Z-ZF gave comments on metabolomics and wrote the manuscript. All authors contributed to the article and approved the submitted version.

## Conflict of Interest

X-YS and MH were employed by company RSKT Biopharma Inc., Dalian, Liaoning, China. The remaining authors declare that the research was conducted in the absence of any commercial or financial relationships that could be construed as a potential conflict of interest.

## References

[1] Kastelein JJ van der Steeg WA Holme I Gaffney M Cater NB Barter P. Lipids, apolipoproteins, and their ratios in relation to cardiovascular events with statin treatment. Circulation. (2008) 117:3002–9. 10.1161/CIRCULATIONAHA.107.713438 18519851

[2] Budoff M. Triglycerides and triglyceride-rich lipoproteins in the causal pathway of cardiovascular disease. Am J Cardiol. (2016) 118:138–45. 10.1016/j.amjcard.2016.04.004 27184174

[3] Vallejo-Vaz AJ Corral P Schreier L Ray KK. Triglycerides and residual risk. Curr Opin Endocrinol Diabetes Obesity. (2020) 27:95–103. 10.1097/MED.0000000000000530 32073428

[4] Wright AK Kontopantelis E Emsley R Buchan I Mamas MA Sattar N. Cardiovascular risk and risk factor management in type 2 diabetes mellitus. Circulation. (2019) 139:2742–53. 10.1161/CIRCULATIONAHA.118.039100 30986362

[5] Vergès B. Pathophysiology of diabetic dyslipidaemia: where are we? Diabetologia. (2015) 58:886–99. 10.1007/s00125-015-3525-8 25725623 PMC4392164

[6] Standards of Medical Care in Diabetes-2019. Abridged for primary care providers. Clinical Diabetes. (2019) 37:11–34. 10.2337/cd18-0105 30705493 PMC6336119

[7] Tada H Kawashiri MA Nomura A Yoshimura K Itoh H Komuro I. Serum triglycerides predict first cardiovascular events in diabetic patients with hypercholesterolemia and retinopathy. Eur J Prevent Cardiol. (2018) 25:1852–60. 10.1177/2047487318796989 30160521

[8] Fan W Philip S Granowitz C Toth PP Wong ND. Residual hypertriglyceridemia and estimated atherosclerotic cardiovascular disease risk by statin use in U.S. Adults with diabetes: National Health and Nutrition Examination Survey 2007–2014. Diabetes Care. (2019) 42:2307–14. 10.2337/dc19-0501 31575639

[9] Hess CN Low Wang CC Hiatt WR. PCSK9 Inhibitors: mechanisms of action, metabolic effects, and clinical outcomes. Annual Rev Med. (2018) 69:133–45. 10.1146/annurev-med-042716-091351 29095667

[10] Third Report of the National Cholesterol Education Program (NCEP). Expert panel on detection, evaluation, and treatment of high blood cholesterol in adults (Adult Treatment Panel III) final report. Circulation. (2002) 106:3143–421. 10.1161/circ.106.25.3143 12485966

[11] Oh RC Trivette ET Westerfield KL. Management of hypertriglyceridemia: common questions and answers. Am Family Physician. (2020) 102:347–54. 32931217

[12] Nichols GA Philip S Reynolds K Granowitz CB Fazio S. Increased residual cardiovascular risk in patients with diabetes and high versus normal triglycerides despite statin-controlled LDL cholesterol. Diabetes Obesity Metabolism. (2019) 21:366–71. 10.1111/dom.13537 30225881 PMC6587847

[13] Reiner Z. Managing the residual cardiovascular disease risk associated with HDL-cholesterol and triglycerides in statin-treated patients: a clinical update. NMCD. (2013) 23:799–807. 10.1016/j.numecd.2013.05.002 23932901

[14] Ezad S Cheema H Collins N. Statin-induced rhabdomyolysis: a complication of a commonly overlooked drug interaction. Oxford Med Case Rep. (2018) 2018:omx104. 10.1093/omcr/omx104 29593874 PMC5853001

[15] Ward NC Watts GF Eckel RH. Statin toxicity. Circulation Res. (2019) 124:328–50. 10.1161/CIRCRESAHA.118.312782 30653440

[16] Shah SH Kraus WE Newgard CB. Metabolomic profiling for the identification of novel biomarkers and mechanisms related to common cardiovascular diseases: form and function. Circulation. (2012) 126:1110–20. 10.1161/CIRCULATIONAHA.111.060368 22927473 PMC4374548

[17] Shou J Chen PJ Xiao WH. The Effects of BCAAs on insulin resistance in athletes. J Nutritional Sci Vitaminol. (2019) 65:383–9. 10.3177/jnsv.65.383 31666474

[18] Siddik MAB Shin AC. Recent progress on branched-chain amino acids in obesity, diabetes, and beyond. Endocrinol Metabolism. (2019) 34:234–46. 10.3803/EnM.2019.34.3.234 31565875 PMC6769348

[19] Choi SC Brown J Gong M Ge Y Zadeh M Li W. Gut microbiota dysbiosis and altered tryptophan catabolism contribute to autoimmunity in lupus-susceptible mice. Sci Translational Med. (2020) 12:eaax2220. 10.1126/scitranslmed.aax2220 32641487 PMC7739186

[20] Zheng Y Hu FB Ruiz-Canela M Clish CB Dennis C Salas-Salvado J. Metabolites of glutamate metabolism are associated with incident cardiovascular events in the PREDIMED PREvención con DIeta MEDiterránea (PREDIMED) trial. J Am Heart Association. (2016) 5:e003755. 10.1161/JAHA.116.003755 27633391 PMC5079035

[21] Jing L Yun-Feng C Xiao-Yu S Liang H Sai-Nan L Wen-Qing G. Plasma tyrosine and its interaction with low high-density lipoprotein cholesterol for type 2 diabetes mellitus in Chinese. J Diabetes Invest. (2019) 10:491–8. 10.1111/jdi.12898 29999591 PMC6400201

[22] Maltais-Payette I Allam-Ndoul B Pérusse L Vohl MC Tchernof A. Circulating glutamate level as a potential biomarker for abdominal obesity and metabolic risk. NMCD. (2019) 29:1353–60. 10.1016/j.numecd.2019.08.015 31668457

[23] Ottosson F Smith E Melander O Fernandez C. Altered asparagine and glutamate homeostasis precede coronary artery disease and type 2 diabetes. J Clin Endocrinol Metabol. (2018) 103:3060–9. 10.1210/jc.2018-00546 29788285

[24] Li J Wang Y Li H Zuo Z Lin J Wang A. Homocysteine level predicts response to dual antiplatelet in women with minor stroke or transient ischemic attack: subanalysis of the CHANCE trial. Arterioscler Thrombosis Vascular Biol. (2020) 40:839–46. 10.1161/ATVBAHA.119.313741 31941381

[25] Luo HH Feng XF Yang XL Hou RQ Fang ZZ. Interactive effects of asparagine and aspartate homeostasis with sex and age for the risk of type 2 diabetes risk. Biol Sex Differences. (2020) 11:58. 10.1186/s13293-020-00328-1 33092635 PMC7579815

[26] Alberti KG Zimmet PZ. Definition, diagnosis and classification of diabetes mellitus and its complications. Part 1: diagnosis and classification of diabetes mellitus provisional report of a WHO consultation. Diabetic Med. (1998) 15:539–53. 10.1002/(SICI)1096-9136(199807)15:7&lt;539::AID-DIA668&gt;3.0.CO;2-S 9686693

[27] Luo HH Li J Feng XF Sun XY Li J Yang X. Plasma phenylalanine and tyrosine and their interactions with diabetic nephropathy for risk of diabetic retinopathy in type 2 diabetes. BMJ Open Diabetes Res Care. (2020) 8:491–8. 10.1136/bmjdrc-2019-000877 32883686 PMC7473660

[28] Wang Q Sun T Cao Y Gao P Dong J Fang Y. A dried blood spot mass spectrometry metabolomic approach for rapid breast cancer detection. OncoTargets Therapy. (2016) 9:1389–98. 10.2147/OTT.S95862 27042107 PMC4795570

[29] Bagheri M Djazayery A Farzadfar F Qi L Yekaninejad MS Aslibekyan S. Plasma metabolomic profiling of amino acids and polar lipids in Iranian obese adults. Lipids Health Dis. (2019) 18:94. 10.1186/s12944-019-1037-0 30967146 PMC6456979

[30] Hasegawa T Iino C Endo T Mikami K Kimura M Sawada N. Changed amino acids in NAFLD and liver fibrosis: a large cross-sectional study without influence of insulin resistance. Nutrients. (2020) 12:1450. 10.3390/nu12051450 32429590 PMC7284573

[31] Moran-Ramos S Ocampo-Medina E Gutiérrez-Aguilar R Macías-Kauffer L Villamil H López-Contreras B. An amino acid signature associated with obesity predicts 2-year risk of hypertriglyceridemia in school-age children. Sci Rep. (2017) 7:5607. 10.1038/s41598-017-05765-4 28717206 PMC5514079

[32] Wiklund P Zhang X Tan X Keinänen-Kiukaanniemi S Alen M Cheng S. Serum amino acid profiles in childhood predict triglyceride level in adulthood: a 7-year longitudinal study in girls. J Clin Endocrinol Metabol. (2016) 101:2047–55. 10.1210/jc.2016-1053 26967691

[33] Bifari F Nisoli E. Branched-chain amino acids differently modulate catabolic and anabolic states in mammals: a pharmacological point of view. Br J Pharmacol. (2017) 174:1366–77. 10.1111/bph.13624 27638647 PMC5429325

[34] Shimomura Y Obayashi M Murakami T Harris RA. Regulation of branched-chain amino acid catabolism: nutritional and hormonal regulation of activity and expression of the branched-chain alpha-keto acid dehydrogenase kinase. Curr Opin Clin Nutrition Metabolic Care. (2001) 4:419–23. 10.1097/00075197-200109000-00013 11568504

[35] Zhou M Shao J Wu CY Shu L Dong W Liu Y. Targeting BCAA catabolism to treat obesity-associated insulin resistance. Diabetes. (2019) 68:1730–46. 10.2337/db18-0927 31167878 PMC6702639

[36] Mook-Kanamori DO Römisch-Margl W Kastenmüller G Prehn C Petersen AK Illig T. Increased amino acids levels and the risk of developing of hypertriglyceridemia in a 7-year follow-up. J Endocrinol Invest. (2014) 37:369–74. 10.1007/s40618-013-0044-7 24682914 PMC3972444

[37] Leighton E Sainsbury CA Jones GC. A practical review of c-peptide testing in diabetes. Diabetes Therapy. (2017) 8:475–87. 10.1007/s13300-017-0265-4 28484968 PMC5446389

[38] Yamakado M Nagao K Imaizumi A Tani M Toda A Tanaka T. Plasma free amino acid profiles predict four-year risk of developing diabetes, metabolic syndrome, dyslipidemia, and hypertension in Japanese population. Sci Rep. (2015) 5:11918. 10.1038/srep11918 26156880 PMC4496670

[39] Geidenstam N Magnusson M Danielsson A Gerszten R Wang T Reinius L. Amino acid signatures to evaluate the beneficial effects of weight loss. Int J Endocrinol. (2017) 2017:1–12. 10.1155/2017/6490473 28484491 PMC5412138

[40] Litwack G. Chapter 7 - Glycogen and Glycogenolysis. In: Litwack G, editor. Human Biochemistry. Boston: Academic Press (2018). p. 161–81. 10.1016/B978-0-12-383864-3.00007-7

[41] Sattar N Scherbakova O Ford I O'Reilly DS Stanley A Forrest E. Elevated alanine aminotransferase predicts new-onset type 2 diabetes independently of classical risk factors, metabolic syndrome, and C-reactive protein in the west of Scotland coronary prevention study. Diabetes. (2004) 53:2855–60. 10.2337/diabetes.53.11.2855 15504965

[42] Burgert TS Taksali SE Dziura J Goodman TR Yeckel CW Papademetris X. Alanine aminotransferase levels and fatty liver in childhood obesity: associations with insulin resistance, Adiponectin, and visceral fat. J Clin Endocrinol Metabol. (2006) 91:4287–94. 10.1210/jc.2006-1010 16912127

[43] Takashina C Tsujino I Watanabe T Sakaue S Ikeda D Yamada A. Associations among the plasma amino acid profile, obesity, and glucose metabolism in Japanese adults with normal glucose tolerance. Nutrition Metabol. (2016) 13:5. 10.1186/s12986-015-0059-5 26788116 PMC4717594

[44] Balasubramanian MN Butterworth EA Kilberg MS. Asparagine synthetase: regulation by cell stress and involvement in tumor biology. Am J Physiol Endocrinol Metabol. (2013) 304:E789–99. 10.1152/ajpendo.00015.2013 23403946 PMC3625782

[45] Durrington PN Bhatnagar D Mackness MI Morgan J Julier K Khan MA. An omega-3 polyunsaturated fatty acid concentrate administered for one year decreased triglycerides in simvastatin treated patients with coronary heart disease and persisting hypertriglyceridaemia. Heart. (2001) 85:544–8. 10.1136/heart.85.5.544 11303007 PMC1729738

[46] Goldie C Taylor AJ Nguyen P McCoy C Zhao X-Q Preiss D. Niacin therapy and the risk of new-onset diabetes: a meta-analysis of randomised controlled trials. Heart. (2016) 102:198. 10.1136/heartjnl-2015-308055 26370223 PMC4752613

[47] Amend KL Landon J Thyagarajan V Niemcryk S McAfee A. Incidence of hospitalized rhabdomyolysis with statin and fibrate use in an insured US population. Annals Pharmacother. (2011) 45:1230–9. 10.1345/aph.1Q110 21917557

[48] Luo HH Zhao MD Feng XF Gao XQ Hong M Liu ML. Decreased plasma n6: n3 polyunsaturated fatty acids ratio interacting with high C-peptide promotes non-alcoholic fatty liver disease in type 2 diabetes patients. J Diabetes Invest. (2020). 10.1111/jdi.13469. [Epub ahead of print]. 33244871 PMC8264392

