# Supplementary figures and images for: Plasma Amino Acids and Residual Hypertriglyceridemia in Diabetic Patients Under Statins: Two Independent Cross-Sectional Hospital-Based Cohorts
Source: Front Cardiovasc Med. 2021 May 31;8:605716. doi: 10.3389/fcvm.2021.605716 (PMC8200824; doi:10.3389/fcvm.2021.605716)

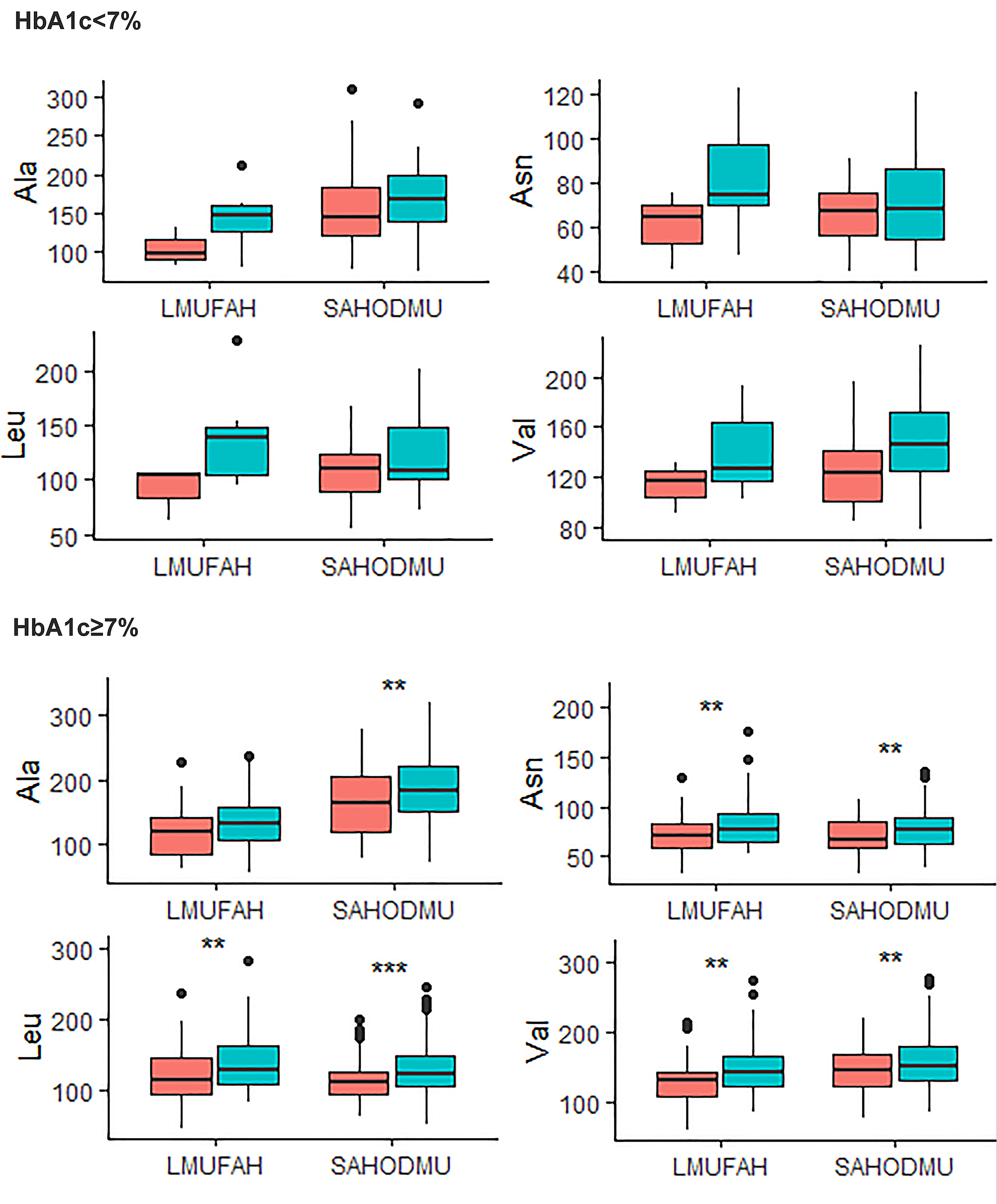

Supplement: Supplementary file 1 [file Image_1.TIF]

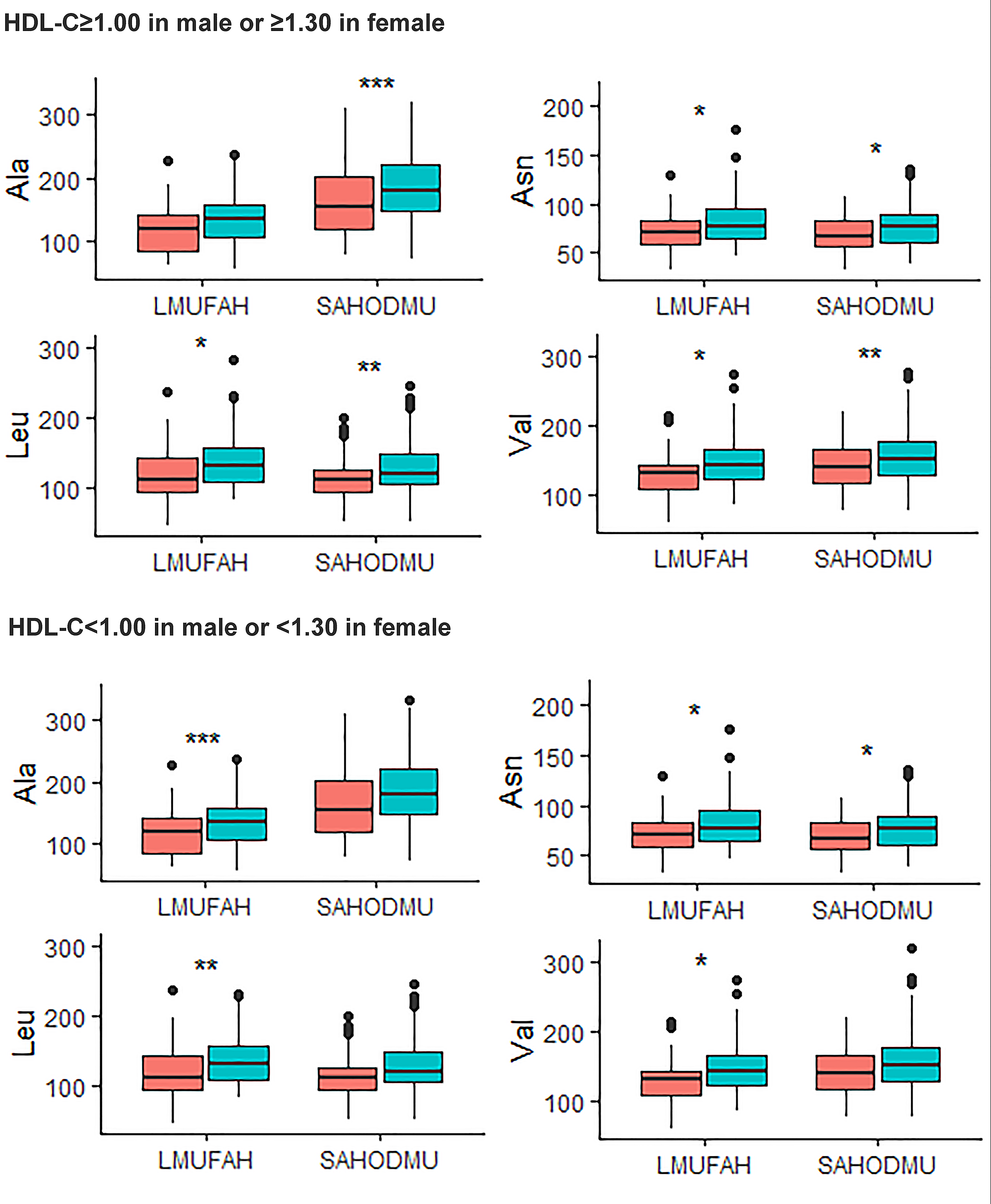

Supplement: Supplementary file 2 [file Image_2.TIF]
