# Supplementary material for: Plasma Amino Acids and Residual Hypertriglyceridemia in Diabetic Patients Under Statins: Two Independent Cross-Sectional Hospital-Based Cohorts
Source: Front Cardiovasc Med. 2021 May 31;8:605716. doi: 10.3389/fcvm.2021.605716 (PMC8200824; doi:10.3389/fcvm.2021.605716)
Supplement: Supplementary file 3 [file Data_Sheet_1.doc]

Table S1. Plasma amino acids levels in diabetic patients without cardiovascular diseases and statins treatment according to TG levels

|  | TG < 1.7 mmol/L | TG ≥ 1.7 mmol/L | P-value |
| --- | --- | --- | --- |
| LMUFAH |  |  |  |
| Ala, mmol/L | 122.00 ± 43.92 | 138.20 ± 53.24 | 0.0039 |
| Asn, mmol/L | 76.41 ± 24.96 | 82.96 ± 24.18 | 0.0190 |
| Leu, mmol/L | 133.10 ± 50.44 | 143.90 ± 44.55 | 0.0462 |
| Val, mmol/L | 139.40 ± 46.85 | 148.10 ± 39.38 | 0.0734 |
| SAHODMU |  |  |  |
| Ala, mmol/L | 159.40 ± 54.19 | 181.30 ± 55.42 | 0.0012 |
| Asn, mmol/L | 71.05 ± 16.80 | 79.25 ± 22.17 | 0.0019 |
| Leu, mmol/L | 117.60 ± 27.94 | 133.30 ± 35.20 | 0.0002 |
| Val, mmol/L | 138.70 ± 33.41 | 155.90 ± 33.77 | <.0001 |

Abbreviations: TG, triglyceride; LMUFAH, Liaoning Medical University First Affiliated Hospital; SAHODMU, the Second affiliated hospital of Dalian Medical University; Ala, aanine; Asn , asparagine; Leu, leucine; Val, valine;

Data are mean ± standard deviation;

P values were derived from independent-samples Student t test and P <0.05 was defined as statistically significant.

Table S2. Associations between amino acids and HTG in subjects without statins treatment

|  | LMUFAH |  |  | SAHODMU |  |  | Pooled |  |
| --- | --- | --- | --- | --- | --- | --- | --- | --- |
|  | OR (95% CI) | P-value |  | OR (95% CI) | P-value |  | OR (95% CI) | P-value |
| Univariable model | | | | | | | | |
| Ala | 1.43 (1.11-1.83) | 0.0046 |  | 1.48 (1.16-1.88) | 0.0016 |  | 1.45 (1.22-1.73) | <.0001 |
| Asn | 1.31 (1.04-1,64) | 0.0203 |  | 1.54 (1.21-1.97) | 0.0006 |  | 1.41 (1.19-1.67) | <.0001 |
| Leu | 1.26 (1.00-1.57) | 0.0481 |  | 1.68 (1.30-2.16) | <.0001 |  | 1.43 (1.20-1.69) | <.0001 |
| Val | 1.23 (0.98-1.53) | 0.0791 |  | 1.66 (1.29-2.13) | <.0001 |  | 1.40 (1.19-1.66) | <.0001 |
| Multivariable model | | | | | | | | |
| Ala | 1.35 (1.04-1.75) | 0.0230 |  | 1.65 (1.24-2.19) | 0.0006 |  | 1.48 (1.22-1.79) | <.0001 |
| Asn | 1.31 (1.03-1.68) | 0.0303 |  | 1.43 (1.07-1.91) | 0.0145 |  | 1.36 (1.13-1.64) | 0.0012 |
| Leu | 1.26 (0.98-1.61) | 0.0677 |  | 1.55 (1.14-2.11) | 0.0051 |  | 1.36 (1.13-1.65) | 0.0015 |
| Val | 1.19 (0.93-1.52) | 0.1652 |  | 1.58 (1.19-2.10) | 0.0015 |  | 1.34 (1.12-1.61) | 0.0018 |

Abbreviations: HTG, hypertriglyceridemia; LMUFAH, Liaoning Medical University First Affiliated Hospital; SAHODMU, the Second affiliated hospital of Dalian Medical University; OR, odds ratio; CI, confidence interval; Ala, Alanine; Asn , Asparagine; Leu, Leucine; Val, Valine;

Logistic regression was used to obtain per standard deviation increased OR;

Multivariable model, adjusted for age, sex, body mass index, duration of diabetes, diabetic nephropathy, high-density lipoprotein cholesterol, low-density lipoprotein cholesterol and glycated hemoglobin;

Figure S1. Plasma amino acids levels according to TG levels in groups with either normal or abnormal HbA1c.

Abbreviations: TG, triglyceride; HbA1c, glycated hemoglobin; LMUFAH, Liaoning Medical University First Affiliated Hospital; SAHODMU, the Second affiliated hospital of Dalian Medical University; Ala, Alanine; Asn , Asparagine; Leu, Leucine; Val, Valine;

Normal HbA1c, HbA1c<7.0%; abnormal HbA1c, HbA1c≥7.0%;

*, P<0.05; **, P<0.01; ***, P<0.001.

Figure S2. Plasma amino acids levels according to TG levels in groups with either normal or abnormal HDL-C.

Abbreviations: TG, triglyceride; HDL-C, high-density lipoprotein cholesterol; LMUFAH, Liaoning Medical University First Affiliated Hospital; SAHODMU, the Second affiliated hospital of Dalian Medical University; Ala, Alanine; Asn , Asparagine; Leu, Leucine; Val, Valine;

Normal HDL-C, HDL-C ≥1.00 in male or ≥1.30 mmol/L in female; abnormal HDL-C, HDL-C <1.00 in male or <1.30 mmol/L in female;

*, P<0.05; **, P<0.01; ***, P<0.001.
